# Reverse evidence based medicine

**DOI:** 10.11604/pamj.2013.16.89.2782

**Published:** 2013-11-10

**Authors:** George Thomas

**Affiliations:** 1 Department of Cardiology, Saraf Hospital, Sreekandath Road, Kochi 682 016, India

**Keywords:** Evidence based medicine, healthcare economics, reverse evidence

## To the editors of the Pan African Medical Journal

Evidence-based medicine may have many deficiencies [1]. But in the absence of any better system, it is the best option for good medical practice. But what do we do when the evidence-based treatment is too expensive for a patient? Here I describe the principle of "reverse evidence" to provide low cost but ethical treatment to a less fortunate patient in India.

A 49 year old male with ischemic heart disease attended our free medical camp conducted on the World Heart Day 2008. He was on metoprolol 50 mg bid, aspirin-clopidogrel 75-75 mg, ramipril 5 mg, simvastatin 20 mg and isosorbide mononitrate 20 mg bid prescribed by a private practitioner. This was an excellent evidence-based treatment for this patient [2]. However he is a daily wage unskilled laborer earning rupees150 (USD 3) per day has no insurance. The cost of medications came to about rupees 50 (USD1) per day. His complaint was that he could not afford the medications. There was no provision for free medicines at the camp.

Like two sides of a coin, all evidences have two sides - obverse and reverse. We tend to follow the obverse side and call it the "evidence" whereas the reverse is also evidence and true. To check the reverse evidence, the raw data of a clinical trial is taken and a commonsense appraisal of the number of patients in the placebo or existing treatment arm is done. If the majority in the comparator arm has favorable outcomes, this will constitute the reverse evidence. This is done without complicated statistical analyses. While the evidence would support the new treatment, the reverse evidence will examine if the placebo or existing treatment has reasonably favorable outcomes. This will be useful in making ethical decisions on the face of the higher costs of the newer treatments.

Here the three expensive medications were ramipril, clopidogrel and simvastatin. We reviewed the evidences for these drugs in the following well-designed randomized controlled trials. In the HOPE study [3] there were 4645 patients in the ramipril group and 4652 patients in the placebo group. 651 patients in the ramipril group and 826 patients in the placebo group had unfavorable outcomes. That means 3994 (86%) patients in the ramipril group and 3826 (82%) patients in the placebo group had favorable outcomes. *Thus the majority in the placebo arm had a favorable outcome and the treatment arm was not devoid of adverse outcomes*. In the landmark 4S study [4] too, the majority in the placebo arm had a favorable outcome. The data in the CAPRIE study [5] also did not prove that clopidogrel was exceptionally better than aspirin. Based on these reverse evidences ramipril, clopidogrel and simvastatin were taken off the prescription.

Now, he is happy and doing well on metoprolol, aspirin and isosorbide dinitrate at a cost of rupees 5 a day! Besides, it has improved his self respect - being empowered to make out-of-pocket purchase of the medicines rather than depend on insurance or doles.

Reverse evidence-based medicine practice needs detailed patient counseling and patient acceptance. In the present case it was easy because the situation was that of a free medical camp where the patient requested a cheaper treatment. We do not deny the statistical benefits of the evidence based treatments. And every patient deserves the best evidence-based treatments. However when cost is a concern, it is worthwhile to consider the reverse evidence of an intervention if it is relatively inexpensive and reasonably acceptable. In this age of increasing healthcare costs, it would also be advisable for guidelines writers to mention the acceptable reverse evidence as an option in cases where the evidence favors an expensive treatment.
